# Impact of Molecular Profiling on Therapy Management in Breast Cancer

**DOI:** 10.3390/jcm13174995

**Published:** 2024-08-23

**Authors:** Flavia Ultimescu, Ariana Hudita, Daniela Elena Popa, Maria Olinca, Horatiu Alin Muresean, Mihail Ceausu, Diana Iuliana Stanciu, Octav Ginghina, Bianca Galateanu

**Affiliations:** 1 OncoTeam Diagnostic S.A., 010719 Bucharest, Romania; flavia.ultimescu@gmail.com (F.U.); maria.olinca@umfcd.ro (M.O.); horatiu.alin.muresean@gmail.com (H.A.M.); 2 Doctoral School of Medicine, “Carol Davila” University of Medicine and Pharmacy Bucharest, 050474 Bucharest, Romania; 3 Faculty of Biology, University of Bucharest, 050095 Bucharest, Romania; diana.stanciu12@s.unibuc.ro (D.I.S.); bianca.galateanu@bio.unibuc.ro (B.G.); 4 Research Institute of the University of Bucharest, University of Bucharest, 050663 Bucharest, Romania; 5 Faculty of Pharmacy, “Carol Davila” University of Medicine and Pharmacy Bucharest, 020956 Bucharest, Romania; 6 Faculty of Medicine, “Carol Davila” University of Medicine and Pharmacy Bucharest, 050474 Bucharest, Romania; mihail.ceausu@umfcd.ro; 7 Faculty of Dental Medicine, “Carol Davila” University of Medicine and Pharmacy Bucharest, 010221 Bucharest, Romania; octav.ginghina@umfcd.ro; 8 Department of Surgery 3, “Prof. Dr. Al. Trestioreanu” Institute of Oncology Bucharest, 022328 Bucharest, Romania

**Keywords:** breast cancer, molecular profiling, targetable mutations, liquid biopsy, therapy management

## Abstract

Breast cancer (BC) remains the most prevalent cancer among women and the leading cause of cancer-related mortality worldwide. The heterogeneity of BC in terms of histopathological features, genetic polymorphisms, and response to therapies necessitates a personalized approach to treatment. This review focuses on the impact of molecular profiling on therapy management in breast cancer, emphasizing recent advancements in next-generation sequencing (NGS) and liquid biopsies. These technologies enable the identification of specific molecular subtypes and the detection of blood-based biomarkers such as circulating tumor cells (CTCs), circulating tumor DNA (ctDNA), and tumor-educated platelets (TEPs). The integration of molecular profiling with traditional clinical and pathological data allows for more tailored and effective treatment strategies, improving patient outcomes. This review also discusses the current challenges and prospects of implementing personalized cancer therapy, highlighting the potential of molecular profiling to revolutionize BC management through more precise prognostic and therapeutic interventions.

## 1. Introduction

Breast cancer (BC), the most commonly occurring cancer in women and the primary cause of cancer-related deaths among women globally, has a wide range of histopathological aspects, metastatic patterns, genetic polymorphism, prognostic outcomes, and responses to therapy [1,2,3]. BC has traditionally been classified based on clinic-pathological features such as tumor size, stage, and nodal involvement, leading to the identification of multiple BC molecular subtypes based on hormone receptors (estrogen—ER and progesterone—PR) and HER2 status [4,5]. However, these conventional variables alone are insufficient for tailoring individualized therapies. The advent of personalized medicine, through the integration of advanced molecular profiling technologies with traditional clinical and pathological approaches, has significantly enhanced our understanding of the molecular heterogeneity of breast cancer. This integration has also highlighted the variability in treatment responses among patients [6,7]. Consequently, identifying specific molecular subtypes of breast cancer is crucial for developing more personalized and effective treatment strategies, utilizing targeted therapies to improve patient outcomes.

**Figure 1:**
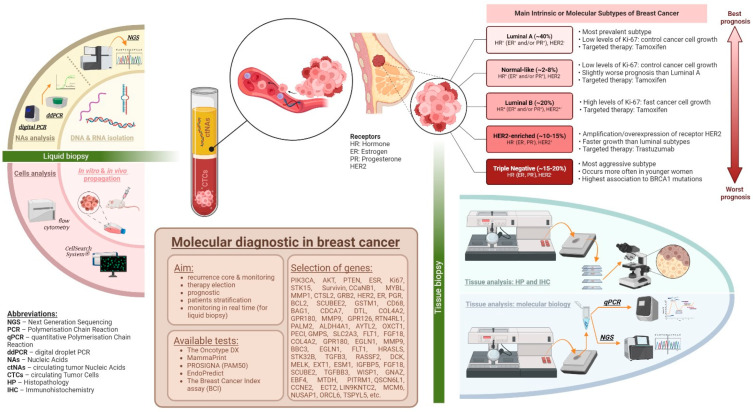
Overview of molecular diagnostics in breast cancer, including liquid biopsy methods, tissue biopsy analysis, and classification of main intrinsic subtypes by prognosis.

This review sums up the current and forthcoming challenges to implementing personalized cancer therapy, highlighting developments in breast cancer molecular profiling and assessing recent and future implementations of next-generation sequencing (NGS) and liquid biopsies to better monitor blood-based biomarkers such as circulating tumor cells, circulating tumor DNA, and circulating tumor educated platelets. An overview of the review is presented in Figure 1.

## 2. Current Approaches in Diagnosis and Therapeutic Management of Breast Cancer

In the assessment of BC, clinical and pathological factors such as tumor diameter, histologic grade, lymph node invasion, the expression of estrogen (ER) and progesterone (PR) receptors, and human epidermal growth factor receptor 2 (HER2) overexpression are regularly included within breast tumor evaluations. However, these conventional assessment criteria may not reliably identify low-risk BC patient categories who may not gain an advantage from chemotherapy in a neoadjuvant setting or extended endocrine therapy. As a result, surrogate biomarker predictive tests for early-stage BC have been designed to accurately predict cancer risk of recurrence and allow medical professionals to make more accurate and consistent treatment selections. These tests assist in the selection of optimal chemotherapy regimens, the minimization of therapy-related side effects, and the avoidance of unrequired treatment.

### 2.1. Immunohistochemistry in Molecular Profiling of Breast Cancer and Therapy

During the therapeutic evaluation of malignancies, immunohistochemistry (IHC) is utilized to identify and quantify the expression level of prognostic biomarkers such as ER, PR, and HER2. In 2013, the St. Gallen International BC Consensus Conference [8] developed a classification system that combines biological tumor markers into surrogate molecular subtypes to facilitate molecular subtyping in the clinical management of BC. This classification system provides more precise information on patient-specific outcomes, risk of relapse, and prospects of achieving a pathological complete response. With a better understanding of the molecular profile of breast tumors, BC patients can receive personalized therapy and eligible patients can be identified for neoadjuvant therapy or more thorough follow-up to monitor the risk of relapse. As a result, immunohistochemistry based on molecular subtyping is currently the predominant approach for predicting tumor responsiveness to hormonal or Trastuzumab treatment.

#### Luminal-Like Breast Cancer Types

Luminal-like breast cancer types make up the majority of breast carcinomas, accounting for 60–70% of all tumors [9]. They have a more favorable prognosis than hormone receptor (HR) negative breast tumors and are generally less responsive to cytotoxic chemotherapy, as indicated by the low rates of pathological complete response seen following neoadjuvant chemotherapy. They have a more favorable prognosis than hormone receptor (HR) negative breast tumors and are often less sensitive to chemotherapy, as evidenced by the low percentages of complete pathological responses reported following neoadjuvant chemotherapy.

Breast tumors classified as luminal A, which account for 40% of all breast cancers [9], exhibit ER overexpression while HER2 and proliferation genes are under-expressed. In both neoadjuvant and metastatic settings, these tumors respond well to endocrine therapy but have a lower response rate to cytotoxic treatment. Luminal B breast cancers, comprising approximately 20% of all breast cancers [9], carry a higher risk of recurrence and exhibit lower expression of ER, variable expression of a HER2, and higher expression of proliferation genes. The HER2-enriched cancer subtype is a subtype that accounts for 12–20% of all BC. They are distinguished by HER2/neu proliferation gene overexpression and low ER expression, presenting a worse prognosis in clinical terms than luminal A tumors.

Basal-like breast cancer subtype accounts for ~15% of all BC and exhibits similar gene expression to normal basal epithelial cells [9]. These tumors frequently lack ER, PR, and HER2 expression, but do express cytokeratin 5/6 and 14 and/or the EGFR (HER1) on IHC stains. Additionally, a high prevalence of BRCA [8] mutations and higher genomic instability, proliferation rate, and histopathologic grade define this subtype [10].

Based on gene expression data, four subtypes have been described: basal-like 1 and basal-like 2 (which differ in immune response), mesenchymal, and luminal AR (androgen receptor). These subtypes have different survival patterns and different levels of sensitivity to neoadjuvant chemotherapy. When RNA and DNA profiling analyses are combined, four distinct subtypes are recognized: luminal AR, mesenchymal, basal-like immunosuppressed, and basal-like immune-activated. Each subtype has different treatment targets (for example, AR and the cell surface mucin EMA (MUC1) in the luminal AR subtype) and prognosis (for example, the basal-like immune-activated subtype has a better prognosis than the basal-like immunosuppressed subtype) [11].

The Claudin low (CL) breast cancer subtype accounts for 5% of all breast cancers and has a unique gene expression profile that distinguishes it from other triple-negative subtypes, negative for ER, PR, HER2, claudin 3, claudin 4, claudin 7, and E-cadherin on IHC testing. This profile is associated with a high risk of recurrence and poor overall survival (OS). Claudin-low breast cancer is also characterized by its high expression of genes associated with immune system function and inflammation, which may contribute to the abundance of lymphocytic infiltration seen in these tumors. This subtype is also associated with high levels of cancer stem cells, which are thought to be involved in tumor initiation, metastasis, and resistance to therapy [12]. Due to its unique gene expression profile and clinical features, Claudin-low breast cancer may require a different treatment approach than other subtypes of breast cancer. Targeted therapies are currently being developed to specifically target the vulnerabilities of this subtype, such as drugs that inhibit the epithelial-to-mesenchymal transition (EMT).

### 2.2. Gene Expression Profiling in Breast Cancer and Its Impact on Therapy Selection

Gene expression profiling (GEP) challenges current approaches to breast cancer classification and treatment, having a significant impact on our understanding of breast cancer heterogeneity at the genomic level. DNA microarray analysis has allowed researchers to measure the expression levels of multiple genes and use this information to construct gene expression profiles for different types of breast cancer. These gene expression patterns are a significant prognostic marker as they enable the assessment of the probability of a patient with newly diagnosed breast cancer to survive, benefit from treatment, or experience a cancer relapse. Aside from extending disease-free and OS, selecting the appropriate therapies based on a patient’s genetic profile is essential for minimizing treatment-related complications. Worldwide, a variety of tumor profiling tests are currently available, and all of them qualify as prognostic biomarkers since they evaluate the risk of recurrence and offer prognostic information in addition to those supplied by common clinical and pathologic variables.

The Oncotype DX assay utilizes PCR to analyze 21 genes and generate a recurrence score (RS), which is used to determine if a woman should undergo chemotherapy after breast cancer treatment. The panel consists of 21 genes, out of which 16 are related to cancer, while the remaining 5 are used as internal controls or reference genes (ACTB, GADHP, RPLPO, GUS, and TFRC). The cancer-related gene set includes genes that are known to have fundamental tumorigenic functions such as cell proliferation (Ki67, STK15, Survivin, CCaNB1, and MYBL), invasion (MMP1 and CTSL2), HER (GRB2 and HER2), estrogen (ER, PGR, BCL2, and SCUBEE2), and other cancer-related genes (GSTM1, CD68, and BAG1) [13]. Oncotype DX generates a score between 0 and 100 that represents the risk of recurrence (RS). If the result is less than 18, the risk of distant recurrence is low, and the claim is that chemotherapy has little or no effect on patient outcomes. A score between 18 and 30 is considered a moderate risk of recurrence and predicts that chemotherapy does not significantly impact patient outcomes. A result of 31 or more is considered a high risk of recurrence and is said to have the capacity to predict the large effect that chemotherapy has on patient outcomes [14]. While the Oncotype DX test is an important tool for guiding BC treatment, recognizing and understanding the size and implications of its errors are essential for optimal clinical use. Currently, the validation of Oncotype DX is limited to HR-positive breast cancer subtypes, and its usefulness for other subtypes of breast cancer remains unclear. There are currently no data clearly demonstrating the clinical utility of Oncotype DX in women with lymph node-positive BC. Furthermore, the false negative rate for HER2 is relatively high, which could result in an underestimation of error risk [15]. Errors in the RS can lead to misclassification, potentially resulting in over-treatment or under-treatment. Discordance in Oncotype RS might occur frequently in cases of bilateral (47%) or unilateral multifocal/multicentric (53%) BC, potentially due to differences in histological grade or the Ki67 proliferating index. Oncologists may have to take into account other clinical and pathological factors to provide the appropriate clinical recommendations for each patient [16].

MammaPrint is a gene expression assay that serves as an independent prognostic marker to predict tumor recurrence in newly diagnosed breast cancer patients, irrespective of conventional clinical and pathologic factors such as tumor diameter, HR, and HER2 status. The test is designed for women with breast cancer in stage I or II that is more than 5 mm, has no regional lymph node metastasis (or micrometastasis <2 mm), and is estrogen receptor-positive and HER 2-negative. The test consists of a panel of 70 genes involved in metastasis and invasion that are implicated in different biological processes such as metastasis and invasion (CDCA7, DTL, COL4A2, GPR180, MMP9, GPR126, RTN4RL1, DIAPH3CDC42BPA, and PALM2), angiogenesis (ALDH4A1, AYTL2, OXCT1, PECI, GMPS, SLC2A3, FLT1, FGF18, COL4A2, GPR180, EGLN1, and MMP9), the avoidance of programmed cell death (BBC3, EGLN1, FLT1, HRASLS, and STK32B), resistance to inhibitory signals (TGFB3, RASSF2, DCK, MELK, and EXT1), self-supply of growth factors (ESM1, IGFBP5, FGF18, SCUBE2, TGFBB3, WISP1, GNAZ, EBF4, MTDH, PITRM1, and QSCN6L1), genes with unrestricted potential for proliferation (CCNE2, ECT2, LIN9KNTC2, MCM6, NUSAP1, ORCL6, TSPYL5, RUNDC1, PRC1, RFC4, and RECQL5), other genes (LGP2, NMU, UCHL5, JHDM1D, AP2B1, MS4A7, and RAB6B), or genes with unknown functions (LOC100288906, C9orf30, ZNF533, LOC730018, and LOC100131053.AA555029) [17]. Based on the test results, the expression profile of the tumor sample is then stratified into low-risk (eligible for hormonotherapy) or high-risk categories (eligible for chemotherapy) for assessing the recurrence of distant metastases within 5 years and the best therapeutic approach. Regarding test limitations, MammaPrint is limited in its ability to be extensively employed in clinical settings since the examined sample must be fresh tissue or fresh frozen tissue. MammaPrint tests cannot be performed on tumor samples that have been embedded in wax and kept in a formaldehyde solution. Additionally, the quick deterioration of the fresh samples may have a severe impact on subsequent procedures like IHC, DNA, and RNA isolation. Differences in RNA extraction, reverse transcription, and microarray or sequencing processes can lead to inconsistent results. Ensuring standardized procedures across different testing sites is important to maintain reliability and avoid potential errors generated by technical variability.

PROSIGNA (PAM50) is also a molecular tumor profiling test that assesses the utility of chemotherapy as an adjunct to hormonal therapy in ER-positive and HER2-negative BC, eligible for patients with unilateral tumors, tumor sizes ranging from 5 mm to 5 cm (stage T1b-T3), ER/PR-positive and HER2-negative, and lymph node without metastasis (or micrometastasis up to 2 mm/or up to 3 lymph node involvement). The panel of 50 genes (ACT3B3, ANLN, BAG1, BCL2, BIRC5, BVLRA, CCNBI, CEP55, EGFR, CXXC5, CCNEI, CDC20, CDC6, CDCA1, CDH3, CENPF, ERBB2, ESR1, EXO1, FGRF4, FOXA1, KRT14, KNTC2, KIF2C, MAPT, KRT5, KRT17, TYMS, FOXC1, GPR160, GR87, HSP150 (UBE2T), MDM2, MELK, MIA, ORC6L, NATI, MYC, MYBL2, MMP11, SLC39A6, SFRP1, RRM2, PTTG1, PHGDH, PGR, MK.167, MLPH, TMEM45B, and UBEC2C) [18] is used to calculate a risk of recurrence score (ROR), which ranges from 0 to 100. This test is suitable for patients with BC who have a unilateral tumor with a size between 5 mm and 5 cm (stage T1b-T3), are positive for hormone receptor (ER/PR) but without HER2 amplification, and have no lymph node involvement or micrometastasis (less than 2 mm in size) or up to three positive lymph nodes.

The ROR score predicts the probability of cancer recurrence in the next 10 years, with a score of 1–10 indicating a 0% chance of recurrence, and scores approaching 61–70 and 91–100 indicating a higher risk of recurrence at around 15% and 33.3%, respectively. Prosigna (PAM50) has been shown in multiple studies to be a reliable predictor of breast cancer survival, irrespective of conventional clinical and pathological risk factors such as lymph node status, HR expression, or tumor grade [19]. As a result, it has been found to reduce chemotherapy eligibility by 44% [20].

The test is exclusively intended for use on FFPE breast cancer tissue specimens from surgical resections and it is not feasible for needle biopsy samples or fresh/frozen breast cancer tissue. The effectiveness of different treatment regimens or patient groups, other than postmenopausal women with tumors between 5 mm and 5 cm and hormone receptor-positive that are lymph node-negative (or micrometastasis <2 mm/or up to 3 lymph nodes positive), has not been yet demonstrated. Therefore, patients with more than three positive lymph nodes and ductal carcinoma in situ (DCIS) are not suitable for testing with Prosigna. Regarding additional constraints of the examination, PAM50 depends on a considerable number of genes, which may elevate both costs and complexity in research. Some studies have reported varying levels of discordance between IHC and PAM50 results. Hee Kyung Kim et al. found up to 38.4% discordance between the IHC subtype and the PAM50 intrinsic subtype [21]. Discordance can lead to different treatment recommendations, as PAM50 and IHC-based classifications can suggest different therapeutic approaches. Errors in classification can also affect prognosis, as each subtype is associated with distinct outcomes and risk profiles [21].

EndoPredict is a gene expression assay intended to optimize and personalize treatment following surgery, assess endocrine and chemotherapy therapy benefits, and estimate the risk of distant recurrence within 10 years. The test is eligible for patients with unilateral tumors, tumor sizes ranging from 5 mm to 5 cm (stage T1b-T3), ER/PR positive, HER2 negative, and lymph nodes without metastasis (or micrometastasis up to 2 mm/or up to 3 lymph node involvement). The EndoPredict test is an RNA-based real-time reverse transcription PCR assay that evaluates the expression of 12 genes, including breast cancer-related genes such as proliferation genes (BIRC5, UBE2C, and DHCR7) and hormone receptor genes (STC2, AZGP1, IL6ST, RBBP8, and MGP), as well as normalization genes (RPL37A, OAZ1, and CALM2) and a control gene for DNA contamination (HBB) [22]. The test generates an EndoPredict score (EP) on a scale of 0 to 15, categorizing patients as low risk (EP between 0 to less than 5) or high risk (EP between 5 and 15). An EPclin Risk Score can also be calculated using a mathematical algorithm that incorporates the activity of these 12 genes along with clinical and pathological factors like tumor size and lymph node status. The EPclin Risk Score ranges from 1 to 3.5 for low risk and >3.5 to 6.0 for high risk at 10 years for distant recurrence [23]. Patients in the high-risk category may benefit from chemotherapy, while those in the low-risk category may avoid unnecessary chemotherapy and its associated side effects. The assay can be performed only for tissue samples from FFPE or a core needle biopsy. Performance characteristics for the test have not been established in patients who received systemic treatment or had localized radiation before surgery or the recurrence of previous primary breast cancers. Samples that are non-invasive BC such as in situ carcinoma (ductal carcinoma in situ), HER2-positive and hormone receptor-negative tumors, or tumors sized larger than 5 cm are also not suitable for testing. Regarding test limitations, some studies have reported high discordance rates between MammaPrint and EndoPredict risk classifications. Jahn et al. found a discordance rate of 44% between tumors classified as high risk by MammaPrint and EndoPredict [24]. Oncologists should be aware of these discrepancies between different tests that could significantly impact treatment decisions, leading to different recommendations regarding the use of adjuvant chemotherapy.

The Breast Cancer Index (BCI) assay is a molecular assay (both prognostic and predictive) that provides personalized information about the individual risk of long-term relapse for patients with hormone receptor-positive breast tumors, allowing clinicians to better assess which patients may benefit from extended adjuvant endocrine therapy. The Breast Cancer Index (BCI) is a gene expression-based test that examines the activity of 11 genes, including an endocrine tumor response biomarker (HOXB13:IL17BR (H/I)), a Molecular Grade Index (MGI) that assesses five proliferative genes (BUB1B, CENPA, NEK2, RACGAP1, and RRM2), and four reference genes (AVTB, HMBS, SDHA, and UBC) [25]. The BCI utilizes these gene expression profiles to predict the potential benefits of endocrine therapy and evaluate an individual’s risk of late recurrence within five years after diagnosis and the cumulative relapse risk within 10 years. Patients are stratified into low-risk and high-risk categories based on these results. Patients with low risk can safely discontinue endocrine therapy after 5 years, while those classified as high risk are advised to continue endocrine therapy for an extended period to avoid unwanted side effects. The BCI is suitable for patients with positive hormone receptors (ER/PR), early-stage tumors (pT1-pT3), no HER2 overexpression, are lymph node-negative (or micrometastasis <2 mm/or up to 3 lymph nodes positive), and have no evidence of metastatic disease. The assay is performed on FFPE tissue and it is not suitable for large tumors (>5 cm) or fresh or fresh frozen tissue. Studies have demonstrated high repeatability and reproducibility for the BCI assay indicating strong reliability. However, errors in the risk score can lead to the misclassification of patients into high-risk or low-risk categories, potentially resulting in over-treatment or under-treatment. For patients with scores within the range of uncertainty, supplementary tests or clinical judgment may be necessary to guide treatment. Moreover, BC often exhibits genomic heterogeneity, with subpopulations of cells displaying different genetic and expression profiles. This heterogeneity can contribute to differences in subtype classification.

Other gene expression profiling tests for breast cancer prediction are available but have yet to be authorized due to insufficient evidence from larger studies to properly assess their clinical utility: Oncotype DX Breast DCIS Score, BluePrint, Breast Cancer Gene Expression Ratio (also known as Theros H/ISM), HERmark Assay, BreastOncPX, BreastPRS, Combimatrix Breast Cancer Profile, DCISionRT, Mammostrat, THEROS Breast Cancer Index, The 76-gene “Rotterdam signature” assay, Insight TNBCtype, MapQuant Dx, NexCourse Breast IHC4, NuvoSelect, SYMPHONY Genomic Breast Cancer Profile, TargetPrint, TheraPrint, and The 41-gene signature assay.

### 2.3. Immune-Related Gene Signatures

Immune-related gene signatures (IRGSs) are specific patterns of gene expression that provide insight into the interactions between the immune system and cancer cells in the tumor microenvironment (TME). These signatures not only help predict patient outcomes but also guide treatment strategies, particularly in the context of immunotherapy. Han et al. proposed an IRS score for BC consisting of 15 genes (LOC220729, SNTN, CYP4F11, ARHGAP39, ATP6V1H, GRHPR, NDRG2, NUP43, HES5, POM121L1P, TNFRSF18, ASAH2, CCR9, NUMA1, and FAM9C) and demonstrated that a high IRS score is associated with shorter OS [26]. Another study found a set of 15 immune-related genes (SLAMF1, TBX21, KLRD1, KLRC4-KLRK1, IL2, KLRK1, ITK, SPN, CD1C, FASLG, CD40LG, CD226, IL7, LAT, and ITGAX) in the TME associated with a favorable prognosis in BC patients [27]. Advancements in immunotherapy, particularly immune checkpoint inhibitors (ICIs) like monoclonal antibodies targeting either the cytotoxic T lymphocyte antigen 4 (CTLA-4)–CD28 or the programmed cell death 1 (PD-1)/programmed cell death 1 ligand 1 (PD-L1) axes, have provided new options for treatment, particularly TNBC or HER2-positive BC. The correlative analysis of the CALGB 40601 and PAMELA trials highlights the significant prognostic and predictive value of IRGS and TILs in early-stage HER2-positive BC [28]. Patients with high IRGS and higher TIL levels were more likely to achieve a pathologic complete response (pCR) to HER2-targeted therapies and improved survival outcomes. However, ICIs are associated with specific side effects known as immune-mediated adverse events that are unique and potentially fatal. Integrating these biomarkers into clinical practice can enhance personalized treatment strategies, improving survival and quality of life for patients with HER2-positive breast cancer. Further research and validation studies are needed to standardize the use of these biomarkers in clinical settings.

The integration of gene expression profiling (GEP) tests into BC management has been a major step forward in the implementation of personalized medicine in clinical practice. By providing detailed insights into the molecular profile of BC, GEP facilitates personalized treatment strategies, predicts treatment responses, provides prognostic information, and guides the development of targeted therapies. This targeted approach to patient selection and therapies accelerates the development and approval of new treatments. Clinical trials designed around specific genetic profiles lead to faster regulatory approval as they play an important role in understanding and translating the insights gained from molecular profiling into effective therapeutic strategies.

Several landmark clinical studies have significantly advanced our understanding of BC treatment and contributed to the development of more effective and personalized treatment strategies. Among them, the TAILORx study has had a substantial impact on clinical practice by demonstrating that patients with early-stage BC, particularly those with low to intermediate recurrence score (RS), can avoid chemotherapy. This clinical trial was designed to guide treatment options for women with HR-positive, HER2-negative, and node-negative BC by using a 21-gene expression assay (Oncotype DX) to assess the risk of cancer recurrence. A total of 10,253 patients with BC were included in the study and stratified according to the RS into low risk (RS between 0–10), intermediate risk (RS between 11–25), and high risk (RS 26 or above). The study results revealed that premenopausal women who are 50 years old or younger and have a recurrence score (RS) of 16–25 may benefit from chemotherapy, while postmenopausal women over the age of 50 may not need chemotherapy if their RS is under 25, indicating the importance of personalized treatment plans according to both age and RS [29].

The MINDACT study is another landmark clinical trial in oncology, designed to assess the effectiveness of the 70-gene MammaPrint assay in guiding adjuvant chemotherapy decisions for women with early-stage BC. The study enrolled 6693 women with early-stage BC, including both node-negative and node-positive (1–3 positive lymph nodes) cases. The main goal was to assess if women with a high clinical risk but low genomic risk could forego chemotherapy without negatively impacting their outcomes. According to the study, women diagnosed with early-stage BC who have a high clinical risk but a low genomic risk of recurrence may not need chemotherapy based on the 70-gene signature. Patients who did not receive chemotherapy had a slightly lower 5-year survival rate without distant metastasis compared to those who did undergo chemotherapy. This provided robust evidence that around 46% of women with BC at high clinical risk may be able to avoid chemotherapy treatment, leading to less over-treatment and fewer side effects [30].

The RxPONDER study is another significant phase III clinical trial designed to evaluate the benefit of chemotherapy in addition to endocrine therapy for women with HR-positive, HER2-negative BC with 1–3 positive lymph nodes and an Oncotype DX recurrence score of 25 or lower. The study enrolled 5083 women and the findings showed that postmenopausal women who have between one and three metastatic axillary lymph nodes and an RS below 25 may opt out of adjuvant chemotherapy without impacting their iDFS. In postmenopausal women, the iDFS rates were very similar between those who received chemotherapy plus endocrine therapy (91.3%) and those who received endocrine therapy alone (91.9%). On the other hand, premenopausal women with one to three positive lymph nodes experienced substantial advantages from chemotherapy, despite having a very low RS. Among premenopausal women, the IDFS rate was 93.9% for the group that received chemotherapy plus endocrine therapy, compared to 89.0% for the endocrine-only group. The addition of chemotherapy led to a 4.9 percentage point improvement in iDFS compared to endocrine therapy alone. Further analyses were conducted to determine if the benefit in premenopausal women was due to ovarian function suppression induced by chemotherapy. However, the results were inconclusive, indicating the need for additional studies to ascertain if ovarian suppression alone could replace chemotherapy in this group [31].

### 2.4. The Synergy between Genomic Testing and Ki-67 in Personalized Breast Cancer Surveillance

Combining genomic tests with Ki-67 levels is useful in personalizing follow-up and monitoring strategies. Ki-67 is a nuclear protein associated with cellular proliferation and an effective marker in the management of BC, providing insights into tumor behavior, guiding treatment decisions, and monitoring therapeutic responses in BC. Patients identified as low-risk by both approaches (molecular and immunohistochemical tests) might require less frequent follow-up, while those with high-risk profiles might benefit from more intensive monitoring to promptly address any recurrence. The ability to predict long-term outcomes based on short-term changes in Ki-67 has been demonstrated by several studies.

POETIC is one of the largest studies trying to assess the predictive value of Ki-67 in determining long-term outcomes in BC. The trial enrolled postmenopausal women diagnosed with early-stage, HR-positive, HER2-negative breast tumors. Participants were randomly assigned to receive either standard care or perioperative endocrine therapy with aromatase inhibitors (AI), administered for two weeks before and after surgery. This brief preoperative treatment aimed to assess early changes in the Ki-67 proliferation index measured from tumor biopsies taken at diagnosis and after two weeks of perioperative therapy. The trial demonstrated that a reduction in Ki-67 levels after two weeks of perioperative endocrine therapy was significantly associated with improved long-term outcomes. Patients exhibiting a substantial drop in Ki-67 had a lower risk of recurrence and better OS rates. Within the HER2-negative subgroup, the 5-year recurrence risk was 4.3% for low–low Ki67, 8.4% for high–-low Ki67, and 21.5% for high–high Ki67 [32].

The WSG-ADAPT is another significant study that explored the utility of Ki-67 as a prognostic and predictive biomarker in early-stage breast BC. The ADAPT trial included patients with early-stage HR+/HER2-BC who were treated with neoadjuvant endocrine therapy (NET) for three weeks. Based on their Oncotype DX Recurrence Score (RS) and on-treatment Ki-67 index, patients were assigned to subsequent adjuvant endocrine therapy or dose-dense neoadjuvant chemotherapy (NAC) followed by adjuvant endocrine therapy. A high Ki-67 index post-NET was associated with a poorer prognosis, whereas a significant reduction in Ki-67 levels indicated a better response to endocrine therapy and a lower risk of recurrence. Specifically, patients with an RS of 0–11 or RS 12–25 with a significant Ki-67 response had 5-year iDFS rates of 93.9% and 92.6%, respectively, indicating that these patients could be spared chemotherapy without compromising their prognosis [33].

### 2.5. Chemotherapy in Premenopausal Women: Evidence-Based Approach

Premenopausal women receiving chemotherapy are at risk of experiencing chemotherapy-induced ovarian failure. The ovarian suppression effect of chemotherapy is a significant concern for premenopausal women undergoing oncological treatment. These effects range from the temporary disruption of menstrual cycles to permanent ovarian failure, impacting fertility and hormonal balance. Chemotherapy drugs (especially alkylating agents like cyclophosphamide, followed by paclitaxel, doxorubicin, or cisplatin) are known to cause direct damage to ovarian follicles. Ovarian damage resulting from chemotherapy occurs through various mechanisms, including direct toxicity to follicles, vascular injury, and oxidative stress. Chemotherapeutic agents harm ovarian follicles by inducing DNA damage and initiating apoptosis in oocytes, which diminishes the ovarian reserve. Additionally, chemotherapy can adversely affect the blood vessels supplying the ovaries, leading to decreased blood flow, follicular atrophy, and subsequent loss of ovarian function. The oxidative stress generated by chemotherapy also damages ovarian tissue, affecting both oocytes and stromal cells and disrupting follicle development. A recent study suggested that chemotherapy drugs can impair ovarian function by inducing ferroptosis due to the excessive accumulation of reactive oxygen species (ROS) [34]. Together, these factors significantly increase the risk of premature ovarian insufficiency and infertility in premenopausal women undergoing chemotherapy. The extent of ovarian damage and the subsequent effect on ovarian function depends on several factors, including the type and dose of chemotherapy, the age of the patient, and their baseline ovarian reserve [35]. Gonadotropin-releasing hormone (GnRHa) analogs have been identified as a potential solution to address these issues and preserve ovarian function during chemotherapy.

The PROMISE-GIM6 trial provides strong evidence supporting the use of the GnRHa triptorelin to prevent chemotherapy-induced premature ovarian insufficiency in premenopausal women with early BC. The use of triptorelin not only helps in preserving ovarian function but also does not compromise the overall efficacy of oncological treatment. Within the GnRHa and control categories, nine and four subjects encountered a pregnancy after therapy, making it a viable option for young women who wish to maintain their fertility post-treatment [36].

The long-term analysis of the POEMS/S0230 trial confirms that the addition of goserelin to chemotherapy not only reduces the risk of premature menopause but also increases the likelihood of post-treatment pregnancy without compromising disease-related outcomes. A higher percentage of patients in the chemotherapy + goserelin group reported having at least one pregnancy compared to those in the chemotherapy-only group (5-year cumulative incidence = 23.1% and 12.2%) [37]. The Anglo-Celtic Group OPTION is another trial that provides robust evidence that goserelin can effectively reduce the incidence of chemotherapy-induced POI and amenorrhea in premenopausal women with early-stage BC, particularly those aged ≤ 40 years [38].

Furthermore, the combined analysis of TEXT (Tamoxifen and Exemestane Trial) and SOFT (Suppression of Ovarian Function Trial) also provided important insights into the long-term effects of adjuvant exemestane with ovarian function suppression (OFS) versus tamoxifen with OFS in premenopausal women with HR-positive BC. Following a 13-year follow-up, the recent evaluation of SOFT-TEXT demonstrated a consistent decrease in recurrence rates with adjuvant exemestane + OFS in comparison to tamoxifen + OFS among premenopausal women with ER/PgR+ BC. Therefore, an aromatase inhibitor should be considered the primary hormonal therapy option for premenopausal women with high-risk characteristics (such as grade 3, T2, and age <35 years) who have HR-positive early BC [39]. Nonetheless, while goserelin can help maintain ovarian function during chemotherapy, it is important to be aware of its potential side effects (which may include hot flashes, mood swings, decreased libido, and loss of bone density).

Given these side effects, cryopreservation emerges as a potential alternative. This procedure is widely recognized as one of the most effective methods for fertility preservation in women of reproductive age who are diagnosed with breast cancer, regardless of the histological type and the hormonal status of the tumor. Recent research indicates that chemotherapy before ovarian tissue cryopreservation does not necessarily impact the success of the procedure. Dolmans et al. reported a 26% pregnancy success rate (including both spontaneous conception and IVF procedures) [40]. They have also demonstrated that while elevated doses of pelvic radiation considerably diminish the chances of achieving a successful pregnancy and may serve as a contraindication, the administration of chemotherapy before ovarian tissue cryopreservation does not always negatively impact the chances of pregnancy (depending on the type of chemotherapy regimen used and the total dosage given).

### 2.6. Epithelial-to-Mesenchymal (EMT) Transition in Breast Carcinoma: Molecular Insights and Therapeutic Targets

EMT in BC is a process that allows epithelial cancer cells to acquire mesenchymal properties, leading to increased invasiveness, motility, and resistance to apoptosis. This transition plays an important role in cancer metastasis and is linked to chemotherapy resistance. This plasticity between epithelial and mesenchymal states is essential for the metastatic spread and establishment of breast cancer cells in new environments [41]. The mechanisms involved in EMT are complex and include EMT-inducing transcription factors (EMT-TFs) (such as Snail, Slug, Twist, and Zeb1/2 [42], which repress epithelial markers and activate mesenchymal markers), signaling pathways (TGF-β pathway, Wnt/β-catenin pathway, and Notch signaling [43]), microRNA regulation [44], epigenetic modifications [45], or cytoskeletal reorganization [46]. Understanding the mechanisms of EMT is essential for developing therapeutic strategies to inhibit BC metastasis by targeting the pathways and regulatory networks involved in this process. Several clinical trials target the epithelial-to-mesenchymal transition (EMT) in BC to address cancer metastasis and drug resistance. Tivantinib, also known as ARQ 197, is a selective inhibitor of the c-MET receptor tyrosine kinase and has shown potential in preclinical and early clinical trials. However, a phase II study of tivantinib in metastatic TNBC showed low response rates, with a 5% response rate [47].

L-NMMA (NG-monomethyl-L-arginine) is an inhibitor of nitric oxide synthase (NOS) and has been studied in clinical trials for its potential in EMT and addressing chemoresistance in BC, particularly TNBC. By inhibiting NOS, L-NMMA alters the tumor microenvironment (making it less supportive of cancer cell survival and spread) and reduces nitric oxide levels, which play a significant role in tumor growth and metastasis. In a Phase II trial, patients with locally advanced TNBC and locally advanced BC had a 45.8% overall response rate [48]. The promising results from these early-phase trials suggest that L-NMMA could be an effective addition to the treatment regimens for TNBC, especially in patients with locally advanced disease. Further research is needed to confirm these findings in larger cohorts.

Disulfiram, a drug primarily used to treat chronic alcoholism, has gained attention for its ability to inhibit EMT and target cancer cells by interfering with key signaling pathways involved in EMT, such as the NF-κB pathway [49]. Ongoing clinical trials are exploring its efficacy in combination with other treatments to enhance its anti-cancer effects. Studies have shown that when disulfiram is used in combination with doxorubicin, it can effectively inhibit the growth of TNBC cells. The use of this combined therapy has been noted to trigger apoptosis and the senescence of tumor cells [50].

### 2.7. Adaptive Molecular Subtyping in Breast Cancer: An Emerging Concept

Unlike the classical approach of intrinsic subtyping, which relies on constant gene expression, adaptive subtyping takes into account the molecular changes that occur after treatment. The concept of “adaptive subtyping” has recently emerged. In the PE-NELOPE-B trial, Denkert et al. demonstrated that the traditional classifications of BC subtypes can change after treatment, frequently transitioning to Luminal B in the advanced stages of the disease [51]. Denkert et al. suggested that comparing matched samples pre- and post-therapy is more effective than the classical subtyping of initial samples, providing a more accurate survival prognosis post-NACT. The study included a total of 1250 patients with BC who were ER-positive and HER2-negative, relapsed after neoadjuvant chemotherapy (NACT), and were randomly assigned to receive either palbociclib or a placebo. The therapy-triggered molecular changes identified provided novel possibilities for organizing BC into adaptive clusters (AC-1–5). Five unique subtypes were distinguished via hierarchical cluster analysis, each with different prognoses for invasive disease-free survival (iDFS): AC1 and AC2 (tumor groups with a favorable prognosis), AC3 and AC4 (tumor groups with a poor prognosis), and the group with the least favorable prognosis belongs to the tumors with a Basal/HER2 + subtype. AC1 and AC3 included tumors that transitioned from Luminal B to Luminal A during NACT, with better iDFS for AC1 while AC2 together with AC4 included tumors that remained Luminal A before and after NACT, with better iDFS for AC2. These findings suggest that adaptive subtyping, which considers changes induced by therapy, provides a more accurate prediction of patient outcomes compared to traditional static subtyping methods [51].

## 3. Molecular Profiling of Breast Cancer and Its Impact on Therapy Management

Breast carcinoma is a heterogeneous disease with complex molecular abnormalities responsible for tumor genesis and development, posing a challenge in the selection of treatment choices and predicting the disease outcomes. Molecular research has allowed an integrated approach to better understand breast cancer heterogeneity and its molecular mechanisms controlling breast cancer progression and response to treatment. Therefore, molecular characterization of BC has provided valuable information about novel cancer driver genes and potentially targetable biomarkers, leading to personalized therapies for BC patients. However, despite the recent advances in molecular research, genetic profiling in routine clinical practice on a wide scale has remained limited. The results of the initial clinical studies revealed that, despite the existence of targetable mutations in the majority of eligible patients, only 11% were treated according to their molecular tumor profile.

The ESCAT (Esmo Scale For Clinical Actionability Of Molecular Targets) has identified the most common molecular alterations associated with breast cancer treatment effectiveness using targetable therapy, including “ERBB2 amplification, germline BRCA1/2 mutations, PI3KCA mutations, microsatellite instability (MSI), and NTRK translocations”. Recognizing the significance of inherited mutations has led to novel strategies for breast cancer prevention and the discovery of targeted therapies. BRCA (Breast Cancer gene) is a tumor-suppressor gene located on chromosome 17q21 (BRCA/1) and chromosome 13q12.3 (BRCA/2), responsible for DNA damage repair, cell cycle control, and genome integrity conservation. Carriers of pathogenic variants of the BRCA genes, mostly with an underlying germline mutation, have a lifetime BC risk ranging from 50 to 85%, with the majority of them having a germline mutation [52]. This served as the starting point for the development of new therapeutic approaches besides conventional chemotherapy, and platinum agents (shown in Table 1) are summarized as the most common agents approved for targeted therapy in BC [53].

PARP inhibitors (polyADP-ribose polymerases) such as Olaparib and Talazoparib have been approved by the Food and Drug Administration (FDA) for metastatic BC and germline BRCA mutation, while Niraparib, Rucaparib, and Veliparib are undergoing clinical trials [54]. These inhibitors have shown improvement in progression-free survival and complete pathological response rate.

The OlympiAD trial highlighted the importance of genetic testing for BRCA mutations in BC patients, thus facilitating the implementation of targeted therapies such as Olaparib. The study enrolled 302 patients with HER2-metastatic breast cancer and germline BRCA (gBRCA) mutations to compare Olaparib with standard chemotherapy in a phase III clinical study. Olaparib has demonstrated significant efficacy in improving (prolonged free survival) PFS and OS in BRCA-mutated BC patients. Olaparib demonstrated a 42% reduction in the risk of disease progression/death, along with a 2.8-month longer median PFS compared to standard therapy. The Olaparib group’s median PFS was 7.0 months, whereas the chemotherapy group’s was 4.2 months. Olaparib’s safety profile matched that of earlier research. When compared to chemotherapy, olaparib’s toxicity profile was more favorable. Upon comparing the Olaparib group to the standard-therapy group, it was observed that the majority of adverse events were either grade 1 or grade 2, with a decrease in the incidence of grade 3 or higher occurrences (36.6% versus 50.5%, respectively). Although there were some limitations to the OlympiAD trial, such as the lack of statistically significant OS improvement and the patients’ heterogeneity concerning HR status and prior use of chemotherapy or platinum-based treatments, the trial overall provided important data supporting the use of Olaparib in BRCA-mutated, HER2-negative mBC (metastatic breast cancer). Additional research is needed to address these limitations and extend the applicability of Olaparib on a larger scale [55].

The PARTNER trial is another important study investigating the efficacy of combining Olaparib with chemotherapy in patients with TNBC who have germline BRCA mutations and those with BRCA wild-type status. The study findings reveal that, despite the effectiveness of Olaparib in treating BRCA-mutated BC, the addition of chemotherapy does not substantially enhance the pathological complete response (pCR) and OS rates when compared to chemotherapy alone in patients with BRCA wild-type status [56].

Talazoparib (Talzenna) is classified as a PARP inhibitor that targets cancer cells with DNA damage repair deficiencies, such as gBRCA. Promising outcomes have been demonstrated in multiple clinical trials, especially in individuals with advanced BC-carrying gBRCA mutations. The EMBRACA trial, a phase III study, demonstrated that Talazoparib significantly increases PFS (8.6 months) in comparison to conventional chemotherapy (5.6 months), resulting in a 46% decrease in the risk of disease progression [57]. Talazoparib is also currently under investigation for its potential to be used in combination with a variety of other drugs to improve effectiveness, address resistance mechanisms, and decrease the number of adverse effects. Several trials are currently ongoing. Among them is the TALAVE trial that combines Talazoparib with Avelumab, an anti-PD-L1 immunotherapy, in patients with advanced BC. The trial aims to evaluate the safety, efficacy, and immunomodulatory effects of this combination. Early findings suggest that PARP inhibitors like Talazoparib can enhance the tumor microenvironment’s susceptibility to immunotherapy [58]. Another study evaluated the combined effect of using a CDK 4/6 inhibitor (Abemaciclib) and Talazoparib, demonstrating the significant inhibition of triple-negative breast cancer cell proliferation by activating apoptotic pathways and inducing G0/G1 cell cycle arrest. These initial findings suggested that the simultaneous administration of Abemaciclib and Talazoparib could enhance the treatment efficacy for TNBC, especially those with BRCA mutations and retinoblastoma protein (RB) deficiencies, potentially addressing resistance issues linked to PARP inhibitors [59].

One of the most promising developments in PARP inhibitor therapy is Saruparib (AZD5305), a next-generation PARP1-selective inhibitor with less toxicity and increased safety and clinical tolerability. Saruparib selectively targets PARP1, which reduces toxicity while preserving efficacy, unlike first-generation PARP inhibitors that target both PARP1 and PARP2. In the phase I/II PETRA trial, Saruparib demonstrated an objective response rate (ORR) of 48.4% and a PFS of 9.1 months among patients with homologous recombination repair-deficient (HRR)-advanced b BC. The safety profile of Saruparib was favorable, with fewer dose reductions and adverse events compared to first-generation PARP inhibitors. Although the results are encouraging, the study is limited by its single-arm design and the limited size of its sample [60].

Research on other PARP inhibitors such as Niraparib, Rucaparib, and Veliparib is also important in advancing targeted therapies for BC, particularly for those with genetic predispositions such as BRCA mutations. These drugs exploit the DNA repair weaknesses in cancer cells, leading to tumor cell death, which is particularly effective in BRCA-mutated BC. The TOPACIO/KEYNOTE-162 trial has shown that combining Niraparib with Pembrolizumab offers a promising therapeutic option for patients with advanced TNBC, especially those with BRCA mutations, along with a tolerable safety profile, warranting further investigation [61].

There are several active clinical studies investigating Rucaparib at the moment. In the Phase Ib COUPLET clinical trial, Rucaparib is being used alone and in conjunction with Atezolizumab (an anti-PD-L1 treatment) to investigate the effects of PARP inhibition in patients with TNBC. The combination of Rucaparib and Atezolizumab has shown a manageable safety profile. Preliminary results indicate that this combination therapy shows potential for substantial anti-tumor efficacy, particularly in individuals with BRCA mutations [62] or those displaying elevated levels of loss of heterozygosity (LOH) in BRCA wild-type breast tumors [63].

The SWOG S1416 clinical trial has delivered valuable insights into the utilization of Veliparib for the treatment of advanced TNBC. The results of the trial indicate that combining Veliparib with cisplatin offers a beneficial treatment option for patients with advanced TNBC who have a BRCA-like phenotype, even in the absence of germline BRCA mutations. Patients treated with Veliparib with cisplatin had a significantly higher PFS than those treated with cisplatin alone. These patients had a BRCA-like phenotype, which reflects the abnormalities in DNA repair observed in people with BRCA mutations. Compared to the control cohort, which had a median PFS of 4.2 months, the Veliparib cohort had a median PFS of 5.9 months. These results emphasize the potential use of PARP inhibitors outside of the typical BRCA-mutated population, providing new treatment options for a larger range of patients with BC [64].

Studies indicate that PARP inhibitor therapy also benefits individuals with BRCA somatic mutations, and their efficacy in BC with BRCA somatic mutations is currently under further investigation [65]. These trials highlight the ongoing efforts to explore the efficacy of PARP inhibitors, both alone and in combination with other treatments, to decrease toxicity and extend progression-free survival for patients with BC.

The PIK3CA (Phosphatidylinositol 3-kinase) gene, located on chromosome 3q.26, is one of the most prevalent genetic abnormalities in luminal BC, occurring in approximately 40% of breast tumors [66], and is part of a family of lipid kinases involved in cell division and proliferation. According to the findings of the SOLAR I trial, tumors with PIK3CA mutations benefit from Alpelisib in conjunction with Fulvestrant, making this a treatment option for patients with ER-positive (ER+)/HER2-negative advanced BC. Median progression-free survival (PFS) increased from 5.7 to 11 months with the addition of Alpelisib [67]. However, due to their many side effects and poor solubility, besides Alpelisib, only another four PI3K inhibitors have been approved by the FDA (Idelalisib, Copanlisib, Umbralisib, and Duvelisib) to date. Furthermore, mutations in the genes belonging to the PI3 kinase pathway (PIK3CA, AKT1, and PTEN) in triple-negative BC patients may be treated with AKT inhibitors like Ipatasertib and Capivasertib. Recent evidence suggests that the acquisition of PIK3CA mutations may be associated with resistance to Fulvestrant-based endocrine treatment, indicating that the evaluation of ESR1 and PIK3CA mutation status may become a therapeutic target for Abemaciclib plus Fulvestrant therapy, which has shown to improve OS in a phase III clinical trial (Monarch 2) [68].

### 3.1. Her2 Monoclonal Antibodies: Clinical Impact and Future Directions in Breast Cancer Treatment

HER2 (Human Epidermal Growth Factor Receptor 2), found on chromosome 17q12, is a tyrosine kinase receptor modulating important biological processes such as cellular division, survival, and differentiation. The overexpression of the HER2 protein is a key prognostic biomarker for identifying individuals with BC who may be eligible for HER2-targeted treatment. According to NGS, somatic mutations in HER2 are seen in 2–5% of primary breast tumors [69]. HER2-targeted drugs for BC are categorized based on their mechanisms of action into three main types: monoclonal antibodies (Trastuzumab, Pertuzumab), anti-body-drug conjugates/ADCs (Trastuzumab Emtansine, Trastuzumab Deruxtecan), and small-molecule tyrosine kinase inhibitors/TKIs (Neritinib, Tucatinib, and Lapatinib).

Trastuzumab, commercialized as Herceptin, is a monoclonal antibody that selectively targets the HER2. The HERA study established Trastuzumab as a standard component of adjuvant treatment for HER2-positive early BC in clinical practice, considerably improving patient outcomes. Following an 11-year median follow-up period, random allocation to 1 year of Trastuzumab resulted in a substantial reduction in the probability of disease-free survival (DFS) events and mortality as compared to the observation group. However, administering Trastuzumab adjuvant for two years did not enhance DFS results when compared to a one-year therapy with this medicine [70].

Pertuzumab is a monoclonal antibody that targets the HER2 protein, offering significant benefits in both early-stage and metastatic settings of BC. The PATRICIA study, a Phase II clinical trial, was designed to evaluate the efficacy of combining pertuzumab with high-dose trastuzumab in patients with HER2+ metastatic BC and brain metastases post-radiotherapy. Adding palbociclib to trastuzumab and endocrine therapy significantly improved PFS compared to the control group. Notably, the objective response rate (ORR) in the CNS was 11%, with clinical benefit rates (CBRs) of 68% at 4 months and 51% at 6 months, indicating substantial clinical advantages along with disease stabilization [71]. Additionally, the study explored the combination of palbociclib with trastuzumab and endocrine therapy in patients with HER2+, HR+ advanced BC utilizing PAM50 molecular subtyping [72]. PAM50 subtyping was used to identify which patients might benefit most from the treatment, reiterating the importance of incorporating molecular subtyping into clinical practice to enhance the BC therapeutic approach. Patients with Luminal A/B tumors showed a more pronounced benefit from the combination therapy, demonstrating improved PFS (12 months PFS = 43.7%) compared to those solely receiving trastuzumab and endocrine therapy (12 months PFS = 21.4%).

The CLEOPATRA trial demonstrated that the addition of pertuzumab to trastuzumab and docetaxel significantly improves progression-free and OS (56.5 months compared to 40.8 months in the placebo group) in patients with HER2-positive metastatic BC [73]. The extended follow-up confirmed that the survival benefits persisted at an 8-year follow-up, demonstrating an OS rate of 37% [74].

Margetuximab (Margenza) is another monoclonal antibody designed to target the HER2 that binds to the HER2 receptor on the surface of cancer cells, blocking HER2 signaling pathways that promote tumor growth and survival. The SOPHIA trial demonstrated improved PFS (5.8 months for margetuximab + chemotherapy compared to 4.9 months for trastuzumab + chemotherapy) and a trend towards better OS compared to trastuzumab (21.6 months versus 19.8 months). Based on these results, Margetuximab has received FDA approval for treating HER2-low metastatic BC along with chemotherapy for patients who have received two or more prior anti-HER2 regimens, at least one of which was for metastatic disease [75].

### 3.2. Her2 Antibody–Drug Conjugates: Advances and Future Directions in Breast Cancer Therapy

Trastuzumab Deruxtecan (T-DXd, Enhertu) is an antibody–drug conjugate (ADC) designed to target HER2-expressing cancer cells. It combines the HER2-targeting capabilities of trastuzumab with a potent topoisomerase I inhibitor, which is released inside the cancer cells to induce tumor cell death. The DESTINY-Breast trials have collectively demonstrated the substantial efficacy of trastuzumab deruxtecan in improving outcomes for patients with both HER2-positive and HER2-low metastatic BC. DESTINY-Breast01 established trastuzumab deruxtecan as a potent option for HER2-positive metastatic BC [76]. The DESTINY-Breast03 trial provided evidence that trastuzumab deruxtecan offers significant clinical benefits over trastuzumab emtansine for patients with HER2-positive metastatic BC, establishing it as a more effective second-line therapy with a manageable safety profile [77]. The DESTINY-Breast04 trial demonstrated that T-DXd significantly improves both PFS and OS in patients with HER2-low metastatic BC, regardless of HR status. Based on these results, T-DXd has received FDA approval for treating HER2-low metastatic BC, providing a new therapeutic option for BC patients who were previously considered HER2-negative [78]. The DESTINY-Breast06 trial findings indicate that T-DXd could potentially serve as a novel standard treatment option for patients with HR-positive, HER2-low metastatic BC who have experienced disease progression with endocrine therapy. Patients with HR-positive, HER2-low (IHC 1+ or 2+/ISH-), or HER2-ultralow (IHC > 0 < 1+) metastatic breast cancer exhibited extended PFS and OS with trastuzumab deruxtecan in comparison to conventional chemotherapy. The median PFS was 13.2 months with T-DXd, while it was 6.8 months with standard chemotherapy. Although the OS data were preliminary at the time of the analysis, there was a tendency towards improved OS outcomes with T-DXd when compared with chemotherapy as a standalone treatment [79]. The DESTINY-Breast07 trial’s preliminary results indicate that trastuzumab deruxtecan, alone or combined with pertuzumab, offers substantial clinical benefits for patients with HER2-positive metastatic BC, demonstrating high response rates and extended PFS. The ORR was 76.0% when using Enhertu alone and 84.0% when using Enhertu in conjunction with pertuzumab. The 12-month PFS rate stood at 80.8% for Enhertu monotherapy and 89.4% for the combination of Enhertu and pertuzumab [80].

### 3.3. Her2 Tyrosine Kinase Inhibitors in Breast Cancer Treatment

Neratinib (Nerlynx) is an oral tyrosine kinase inhibitor (TKI) that irreversibly inhibits HER2 and EGFR kinases (epidermal growth factor receptor), specifically designed to target HER2-positive BC. The NALA trial has shown that combining neratinib with capecitabine leads to a significant improvement in PFS when compared to lapatinib plus capecitabine, presenting a viable treatment option for previously treated patients with HER2-positive metastatic BC. As a result of these findings, the FDA has approved the use of Neratinib in combination with capecitabine for advanced or metastatic HER2-positive BC patients who have undergone two or more prior anti-HER2-based treatments in the metastatic setting [81].

Tucatinib (Tukysa) is an oral small-molecule TKKI that selectively inhibits the HER2 receptor, blocking the proliferation of HER2-positive cancer cells and interfering with tumor growth and survival. The HER2CLIMB trial confirmed that tucatinib, in combination with trastuzumab and capecitabine, significantly improves PFS (7.6 months versus 4.9 months for the placebo group) and OS (24.7 months versus 19.2 months for the placebo group) in patients with HER2-positive metastatic BC, including those with brain metastases who often have limited treatment options [82].

Lapatinib (Tykerb) is an oral small-molecule TKI that targets both the HER2 and EGFR pathways. It is primarily used to treat HER2-positive BC, particularly in patients who have progressed on trastuzumab-based therapies. Its ability to inhibit both HER2 and EGFR pathways makes it a versatile option for combination therapies, providing significant clinical benefits in terms of PFS and OS. In March 2007, Lapatinib received its initial FDA approval for use in combination with capecitabine for the treatment of patients with advanced or metastatic HER2-positive BC. In January 2010, the FDA expanded the approval of lapatinib for use in combination with letrozole. This expanded approval is for postmenopausal women with HR-positive metastatic BC that overexpresses the HER2 receptor, where hormonal therapy is indicated. These approvals were based on clinical trials (the EGF100151 Trial [83] and the EGF30008 Trial [84]) demonstrating the efficacy and safety of lapatinib.

Microsatellite instability (MSI) is considered to be exceedingly rare in BC, occurring in fewer than 1% of cases [85]. Therefore, MMR protein evaluation has been considered unsuitable for MSI screening in BC, and the importance of MMR proteins in microsatellite-stable breast tumors has yet to be thoroughly studied. The incidence of NTRK-rearranged tumors is increasing as molecular profiling becomes more widespread, and NTRK fusion may be discovered in a variety of cancer types. In BC, NTRK mutations are virtually always seen in secretory carcinoma, an extremely uncommon triple negative BC subtype defined by an ETV6-NTRK3 gene fusions [86].

The PTEN (Phosphatase And Tensin Homolog Deleted On Chromosome Ten) gene, located on chromosome 10q23, is involved in various cellular processes and is frequently lost in BC cases, leading to uncontrolled signaling pathways and poorer outcomes in some subtypes. PTEN deficiency may be linked with poorer outcomes in hormone-positive (ER/PR+) and HER2-negative or HER2-positive BC [87]. Several clinical studies demonstrate that PTEN may contribute to Trastuzumab-based therapeutic resistance, implying that PTEN may have a predictive role in breast malignancies [88]. Trastuzumab with chemotherapy in a neoadjuvant setting resulted in significantly diminished pCR (complete pathological response) rates in HER2-positive BC patients with low PTEN expression assessed by immunohistochemistry compared to those with elevated PTEN protein levels [89]. PTEN deficiency may also result in PD-L1 overexpression, suggesting that PTEN may play a role in immune-checkpoint inhibitory response [90]. To summarize, PTEN’s potential as a biomarker in BC is encouraging and warrants further assessment.

AKT is a group of serine/threonine-protein kinases (AKT1, AKT2, and AKT3) located at 14q32.33 that modulate metabolism, proliferation, cell survival, growth, and angiogenesis. Dysregulation of this pathway is commonly associated with BC progression and endocrine resistance to therapy. Targeting the AKT pathway with specific inhibitors offers a promising therapeutic strategy, particularly for patients with genetic alterations in the PI3K/AKT/mTOR pathway. Genetic testing is therefore important for identifying these mutations and patient selection for personalized treatments. Capivasertib and Ipatasertib are oral AKT inhibitors that target all three isoforms of AKT (AKT1, AKT2, and AKT3), inhibiting downstream signaling pathways that promote tumor growth and the survival of breast cancer cells. The phase 3 CAPItello-291 Trial demonstrated that capivasertib, combined with fulvestrant, significantly improves PFS (7.3 months versus 3.9 in the placebo group) and shows a favorable safety profile in patients with HR+, HER2-advanced BC after relapse under aromatase inhibitor treatment and prior therapy with CDK4/6 inhibitors. Further research and regulatory reviews will determine its place in clinical practice [91].

Regarding HRD (Homologous recombination deficiency), the failure of a cell to restore damaged DNA double strands via the homologous recombination repair (HRR) pathway is referred to as homologous recombination (HR), a potential biomarker for genomic instability. Genetic alterations in essential homologous recombination genes like BRCA1 and BRCA2 lead to a deficiency in homologous recombination (HR), which can be a target for therapeutic approaches including PARPi inhibitors. The HRR status and score might function as predictive biomarkers of both progression-free survival and OS and can be calculated using multiple gene sequencing [92]. The current difficulty is that there is no universal method of defining, measuring, or reporting the status of HR using diagnostics in the clinical setting.

ESR1 (Estrogen Sensing Receptor 1), located on chromosome 6, codes for the estrogen receptor and is a predictive biomarker for resistance to endocrine therapy in primary and metastatic BC patients. ESR1 mutations are among the most commonly acquired mutations in BC and are linked to shorter progression-free survival as well as resistance to endocrine treatment. Their prevalence is thought to have a low incidence in primary tumors (~1%) but they are more frequently encountered in metastatic settings or endocrine therapy-resistant BC (10–50%) [93]. Selective Estrogen Receptor Degraders (SERDs) represent a promising class of therapeutic agents designed to treat HR-positive BC by targeting and degrading estrogen receptors (ER). The Emerald Trial is a Phase III clinical trial evaluating the efficacy of elacestrant, an oral selective ER degrader (SERD), in treating patients with ER-positive, HER2-negative advanced or metastatic BC. This trial focused on patients whose disease had progressed after treatment with CDK4/6 inhibitors and other endocrine therapies. Elacestrant demonstrated a statistically significant improvement in PFS (3.8 months) compared to standard endocrine monotherapy options such as fulvestrant, letrozole, anastrozole, or exemestane (1.9 months PFS with standard care). Among patients with ESR1-mutated tumors, median PFS reached 8.6 months with elacestrant compared to 1.9 months with standard therapy, indicating a 39% reduction in the risk of disease relapse or death. Also, patients with prior CDK4/6 inhibitor therapy for at least 6 months had better outcomes with elacestrant compared to standard therapeutic protocols. Based on these results, Elacestrant (Orserdu) was granted FDA approval in 2023 for the management of advanced or metastatic BC in postmenopausal women who are ER-positive, HER2-negative, and have ESR1 mutations, following disease progression after at least one round of prior endocrine therapy. Its oral administration and efficacy in endocrine-resistant disease make it a valuable addition to the range of treatments available for BC [94].

Aromatase inhibitor treatment, which targets ESR1 mutations, increased progression-free survival and OS when used in combination with Fulvestrant [95]. The therapeutic implications of ESR1 mutations were demonstrated in a phase 3 clinical trial (PADA-1) in which metastatic hormone receptor-positive (ER/PR+) breast cancer patients received first-line therapy with aromatase inhibitors and Palbociclib. Patients who were given Fulvestrant in combination with Palbociclib before experiencing disease progression had a significantly longer median duration of progression-free survival (11.9 months versus 5.7 months) [96].

Beyond selective ER modulators (SERMs) and orally administered selective ER degraders (SERDs), several innovative classes of drugs are emerging, including complete ER antagonists (CERANs), proteolysis targeting chimeric molecules (PROTACs), and selective ER covalent antagonists (SERCAs). OP-1250 (Palazestrant) is a complete agonist ER receptor (CERAN) that acts as a selective ER degrader (SERD) by inhibiting estrogen-driven transcriptional activity and reducing tumor growth and proliferation in ER+ BC cells. In preclinical models, it has demonstrated favorable pharmacokinetics (oral availability and penetration of the blood–brain barrier) and potent anti-tumor activity [97]. The preliminary results of one trial indicate that the inclusion of OP-1250 alongside ribociclib and alpelisib has been well clinically tolerated, demonstrating a manageable safety profile, potent anti-tumor efficacy, and sustained benefits, with 41% of patients observing a tumor size decrease in advanced and/or metastatic ER+/HER2-BC [98].

SERCAs represent a novel class of therapeutic drugs designed to specifically address ER+ BC by forming a covalent bond with the ER, leading to irreversible inactivation. This permanent ER deactivation provides a feasible method for decreasing the resistance linked to standard endocrine therapy. A phase 2 clinical study found that H3B-6545 had a tolerable safety profile and anti-tumor effectiveness in previously treated ER+ and HER2-metastatic BC patients, including those with ESR1 mutations. The findings are encouraging since SERCAs offer a potential answer to resistance to endocrine treatments, but more clinical trials are needed to properly prove the efficacy and safety of SERCAs in BC in broader patient groups [99].

Proteolysis-Targeting Chimeras (PROTACs) are a new type of therapeutic agent designed to degrade specific proteins selectively. ARV-471 (Vepdegestrant) is an example of a PROTAC developed to target the estrogen receptor (ER) in BC with ER+/HER2- status. The preliminary result of a clinical trial proved to have a CBR rate of 47% in patients previously treated with CDK4/6 inhibitors [100].

Although traditional endocrine therapies such as SERMs and aromatase inhibitors have significantly improved patient outcomes, resistance to these treatments remains a major challenge. These new emerging therapies are expected to offer novel ways of overcoming resistance mechanisms, provide increased therapeutic responses, and improve OS. Ongoing research is needed to properly prove the efficacy and safety of SERCAs, CERANs, and PROTACs in BC in broader patient groups.

RB1 (Retinoblastoma gene) is a gene located on chromosome 13 that inhibits E2F transcription factors and promotes G1/S arrest and growth limitation. RB1 mutations are found in about 3.68% of breast cancer patients and are associated with a worse prognosis [101]. Clinical studies have shown that CDK4/6 inhibitors in combination with endocrine treatment may improve survival in hormone-positive, HER2-negative metastatic breast cancer, but further research is needed to determine the clinical usefulness of targeting RB1 mutations [102].

NF1 (Neurofibromatosis type 1), located on chromosome codes for the neurofibromin protein, plays a role in cell proliferation and differentiation by activating the RAS pathway. The NF1 mutation is assumed to be an independent prognostic factor linked to poor prognosis and resistance to endocrine treatment [101]. It is encountered in around 7% of metastatic hormone-positive/HER2-negative BC [101,103]. The current treatment strategy involves targeting the NF1 mutation with selumetinib (a MEK inhibitor) or Fulvestrant combined with Binimetinib in clinical trials [104]. For the time being, the prognostic significance of NF1 has not yet been completely understood; however, it may become potentially useful in the future by developing an inhibitor for the RAS pathway.

The PALB2 gene (Partner And Localizor of BRCA2), found on chromosome 16, is a gene that modulates DNA repair damage through the homologous recombination repair pathway and interactions with both BRCA1 and BRCA2, maintaining the genome’s integrity. Recent multigenomic testing has shown that PALB2 is a high-risk BC predisposing gene associated with aggressive clinicopathological features such as a triple-negative phenotype and advanced disease stage [105]. Women with PALB2 mutations have an estimated BC risk of 14% at age 50 and 35% at age 70 [106]. Due to the discovery of PALB2 mutations, precision medicine and personalized risk evaluation of PALB2 mutations in BC is now possible, although long-term validation clinical trials are needed. Precision medicine and personalized risk assessment for PALB2 mutation-associated breast cancer are now achievable, but further clinical trials are needed to validate the efficiency of targeting this gene for a therapeutic approach. Clinical trials have demonstrated the efficacy of PARP inhibitors (Talazoparib and Olaparib) and platinum-based chemotherapy in treating BC associated with PALB2 mutations. The detection of PALB2 mutations through genetic testing can assist in personalizing treatment strategies, allowing for the targeted application of these medications in patients with mutated PALB2 BC [107].

CDH1 (E-cadherin gene), situated on chromosome 16q22.1, is a cell-to-cell adhesion molecule and tumor-suppressor protein. Somatic CDH1 inactivation is linked to an aggressive pattern of BC, with lymphovascular invasion and metastases in the axillary lymph nodes, resulting in poorer prognosis and reduced OS [108]. As a result, CDH1 may play a possible role in the clinical care of BC patients as a prognostic biomarker and molecular target for therapeutic approaches [109], primarily through the process of the epithelial-to-mesenchymal transition (EMT). Therefore, targeting the pathways associated with EMT and cell migration may be a potential therapeutic strategy. Recent research suggests that the co-administration of focal adhesion kinase (FAK) inhibitors and ROS1 inhibitors could provide an effective treatment option for patients with CDH1-deficient BC [110].

TP53 (Tumor protein P53), located on chromosome 17, codes for the transcription factor p53, a regulatory protein involved in the regulation of the cell cycle-programmed cellular death, and damaged DNA repair. TP53 is found in 30% [111] of all breast tumors and approximately 80% [112] of triple-negative BC. Breast tumors with TP53 mutations are associated with a worse prognosis due to their increased susceptibility to have a more aggressive biological behavior and be less responsive to chemotherapy and radiation. The relevance of the TP53 mutation as a predictive factor for the effectiveness of chemotherapy, hormone treatment, and PARP inhibitors has been highlighted in recent studies. The presence of TP53 mutations may have clinical consequences; however, the relevance of TP53 mutations as a prognostic biomarker for chemotherapy and radiotherapy effectiveness is still being investigated, with no official therapy recommendations developed yet [113].

CHECK2 (Checkpoint kinase 2), found on chromosome 22q12.1, is a serine/threonine kinase involved in regulating the cell cycle and damaged DNA repair. BC risk is connected to family history. The risk is roughly 20% in carriers who have no afflicted relatives, but it can rise to 44% when both first- and second-degree relatives are affected [114]. CHEK2 mutations not only predispose to BC but can predict the susceptibility of risk of metastasis and can also be used to predict a patient’s response to therapy. For example, c.1100delC and I157T can identify the susceptibility to metastasis [115], and other CHEK2 mutations are associated with resistance to anthracycline-based chemotherapy and response to epirubicin [116]. As a result, CHECK2 may have a possible role in the clinical care of BC patients as a molecular target for the therapeutic approach.

ATM (the Ataxia-telangiectasia mutated gene), located on chromosome 11q23, is an oncosuppressor gene that codes for a protein implicated in cellular growth, apoptosis, gene regulation, and telomere integrity. Heterozygous carriers of these mutations have an increased risk (x3 fold) of developing BC [117]. Although the clinical and pathologic aspects of ATM-associated BC are not well established, it is known that ATM-mutated BCs are typically endocrine-positive, with a more aggressive clinical course, and consequently, have a worse prognosis [118]. In terms of therapeutic considerations, ATM might make cancer cells susceptible to platinum agents, but it has a negative effect when combined with radiation, increasing the chances of developing a second tumor following radiotherapy. Based on these findings, the ATM gene might be a predictive biomarker and a target for therapy [119].

MRE11A (meiotic recombination 11 homolog A), located on chromosome 11, codes for a nuclear protein that plays a role in maintaining the length of telomeres and facilitating homologous recombination. MRE11A is linked to an elevated risk of BC (lifetime risk of 37.2%, three times the normal population risk) [120]. According to current NCCN (National Comprehensive Cancer Network) guidelines, there are insufficient data to establish recommendations for breast MRI (magnetic resonance imaging) and risk-reducing mastectomy (RRM) based only on MRE11A mutant status. However, elevated MRE11 expression was linked to more aggressive clinical course in BC [121]. MRE11 may be a novel oncoprotein and, hence, a potential therapeutic target for BC management.

BRIP1 (Breast Cancer 1 Interacting Helicase 1), located on chromosome 17, is a tumor-suppressor gene that modulates DNA-repairing damage. BRIP1 is mutated in about 2% of breast tumors [122]. When compared to luminal A subtype, basal-like, HER2-enriched, and luminal B subtype BCs have higher levels of BRIP1 expression. According to the NCCN guidelines, there are insufficient data to support therapy management based only on BRIP1 mutation status. To support the clinical value of BRIP1 in determining the prognosis of BC and developing treatment strategies, further investigations are required.

RAD51 is a family of genes (RAD51B, RAD51C, RAD51D, XRCC2, and XRCC3) located on chromosome 17 that encodes a protein involved in HRD and DNA repair. Recent research has linked germline mutations in RAD51C and RAD51D to an increased risk of triple-negative BC. RAD51C mutation carriers may be susceptible to chemotherapeutic treatments [123] and, hence, benefit from medications advised for BRCA1/BRCA2 carriers, such as PARP inhibitors. Targeting the RAD51C gene may be a viable treatment method for the management of breast tumors, but the current NCCN guidelines state that the biological function and predictive significance in BC have yet to be clarified.

STK11 (Serine/threonine kinase 11), located on chromosome 19, is part of the serine/threonine kinase family of proteins and acts as a tumor suppressor. Studies have revealed that mutational inactivation of the gene is an uncommon occurrence and presumably has a small influence on sporadic breast carcinogenesis. Nonetheless, STK11’s relevance in sporadic breast carcinogenesis could not be completely ruled out, and more research is required to assess the involvement of STK11 mutation in breast carcinogenesis.

BARD1 (BRCA1-associated RING domain 1), located on chromosome 2, codes for a protein that binds with BRCA1’s N-terminal region. BARD1 may be of potential interest for innovative therapeutics since it seems to be implicated in the pathophysiology of BC and the processes behind cancer cell chemoresistance [124], enabling the assessment of the mechanisms of drug resistance in breast tumors. Recent studies have demonstrated that the resistance to treatment with Cisplatin and Adriamycin is caused by the overexpression of BARD1 and BRCA1 in tamoxifen-resistant breast carcinoma cells [125]. As a result, BARD1 may be a viable prognostic biomarker for BC prognosis, although more study is needed.

Regarding CASP8 and 3 (Caspase 8 and 3), CASP8 is an initiator caspase that plays a role in the apoptosis of BC cells. The majority of breast tumors overexpress caspase-8 [126], which may one day be used as a predictive biomarker. Caspase-3 expression is linked to poor survival in BC patients and gives additional prognostic values in different phenotypes [127].

CTLA-4 (Cytotoxic T Lymphocyte-Associated Protein-4), situated on chromosome 2, is an immunoregulatory protein that can suppress T-cell activation and reduce the anti-tumoral immune response. CTLA-4 has been reported to be overexpressed in breast tumors [128]. CTLA-4 expression in the tumor microenvironment appears to be related to poorer outcomes and shorter OS [129], being a potential therapeutic target for immunotherapy [130]. More research is needed to validate this finding.

CYP19A1 (Cytochrome P450 Family 19 Subfamily A Member 1), found on chromosome 15, is a member of the cytochrome p450 superfamily and is involved in increasing androgen aromatization in estrogens and catalyzing the last step in estrogen biosynthesis. BC risk and aggressiveness have both been linked to changes in CYP19A1 gene expression levels. Recent research has shown that the main regulatory events for the intratumoral synthesis of estrogens in breast malignant tissues are the expression of high amounts of CYP19A1 and ER as well as higher levels of estrogens [131]. As a result, this enzyme is a molecular target for treatment, particularly in postmenopausal women where hyperestrogenism is the key factor in the emergence and progression of hormone-induced cancers.

FGFR (Fibroblast growth factor receptors) are tyrosine kinase receptors, recently acknowledged as potential risk factors for BC. FGFRs are overexpressed in 14% of BC cases [132]. In triple-negative BC, FGFR1 expression is a poor prognostic predictor [133]. The amplification of FGFR1 is also linked to a poor prognosis in ER-positive malignancies and resistance to endocrine therapy [134]. Increased FGFR3 expression is related to a worse OS rate [135]. FGFR4 facilitates the change from a more differentiated, luminal phenotype to a more aggressive and metastatic, HER2-enriched one [136] in invasive lobular cancer [137] and is implicated in metastasis and endocrine resistance.

H19 (located on chromosome 11p15.5) is a promising potential biomarker for the detection of BC. Recent studies have suggested that H19 overexpression enhances treatment resistance and is linked to poor outcomes in BC patients [138]. The long noncoding RNA H19 quantified in fresh breast biopsies might be a possible biomarker for cancer detection and subcategorization of lesions. Because molecular changes take place before morphological alterations in cancer lesions, the lncRNA H19 might also provide insight into patient prognosis, follow-up, and therapeutical management. Furthermore, low levels of H19 expression in malignant biopsy sample lesions imply higher aggressiveness, whereas high H19 expression indicates less aggressiveness [139].

TERT (Telomerase Reverse Transcriptase) is a gene located on chromosome 5p15.33 that is responsible for encoding the telomerase enzyme, which plays a critical role in maintaining the length of telomeres. In normal breast tissue, telomerase activity is absent, but it is expressed in malignant breast lesions [140]. TERT promoter hotspot mutations and gene amplification are not common in typical breast cancer, but they have been reported in a significant percentage of malignant phyllodes tumors and adenomyoepitheliomas of the breast [141,142]. High levels of telomerase expression in cancer cells are associated with a poor response to therapy, which is why telomerase has become a potential target for cancer treatment [143]. Telomerase-targeted treatment aims to selectively induce apoptosis in cancer cells while minimizing the negative effects on normal cells [143]. Therefore, drugs based on telomerase targeting may be effective against telomerase-positive breast tumors while safeguarding the surrounding healthy cells.

MAP3K1 (Mitogen-Activated Protein Kinase 1) is a serine/threonine located on chromosome 5 that plays a role in cellular migration and survival. The luminal A subtype of breast tumors shows the highest incidence of MAP3K1 mutations. Recent clinical studies have shown that mutations in the MAP3K1 gene in ER-positive BC may be susceptible to targeting by Buparlisib and Letrozole [144].

NBN (Nibrin) is a gene located on chromosome 8q21.3 that is responsible for encoding a protein that is a constituent of the MRE11/RAD50 complex, which is involved in repairing double-strand breaks. Based on preliminary research that has suggested an increased risk for carriers, the NBN gene has been added to breast cancer multigene panels, with the c.657del5 mutation [145] being associated with up to 30% of female breast cancer cases. However, further research is necessary to fully understand the impact of NBN on breast cancer susceptibility.

## 4. Liquid Biopsy as a Modern Tool for the Management of Breast Cancer

Due to the heterogeneity of BC, deciphering its molecular landscape is essential for identifying the best disease management strategy. The molecular signatures, together with the clinical and pathological parameters, impact prognosis and have therapeutic implications, therefore it is crucial to take advantage of all the available tools that could improve the current practice in BC and aid the characterization of the complexity of BC tumors [146]. Among the modern approaches available in oncology, liquid biopsy (LB) has proven to be extremely useful as the use of this technique was intensively accelerated in the last decade for tumor profiling and not only.

Starting with the challenges associated with traditional tissue biopsy, modern approaches shift toward replacing tissue sample analysis with the modern LB, a minimally invasive technique that implies the analysis of tumor-derived components from biological fluids. In BC, tissue biopsy is part of common practice but is still a time-consuming and painful procedure associated with increased treatment costs. Moreover, the tissue sample obtained by biopsy or surgery captures a static snapshot of the tumor and fails to reveal its heterogeneity or evolutionary landscape influenced by time and treatment. Compared with tissue biopsy, LB is a real-time analysis, suitable for capturing intratumor heterogeneity, and can be repeated when needed in cases of patients’ long-term monitoring and therapy personalization [147].

For a long time, the LB was based on the analysis of the following markers from the tumor circulome: circulating tumor cells (CTCs), cell-free nucleic acids (cfNAs), and tumor-derived extracellular vehicles (EVs). However, recent studies have explored the analysis of other tumor-originating components: tumor-educated platelets (TEPs) and a wide spectrum of tumor metabolites and proteins in LB. Tumor cells or tumor-derived components are released in circulation from the primary tumor and/or metastatic deposits, by active secretion, or as a result of necrosis or apoptosis, and are currently explored for early BC detection, staging, prognosis, drug resistance, and minimal residual disease (MRD) monitoring.

### 4.1. Circulating Tumor Cells (CTCs)

For a long time, CTCs were considered tumor cells of epithelial origin that detach from the primary tumor or distal metastasis and intravasate into the bloodstream and/or lymphatic vessels [93]. The detachment process of CTCs is generally spontaneous, but in some cases, the presence of CTCs has also been reported to be the result of the tumor’s mechanical manipulation during surgical interventions [94,95,96,97]. Independent of the detachment trigger, CTCs are considered the culprit of metastasis development, with the detection of cancer cells in the bloodstream being an early sign of disease progression [98].

The metastatic cascade in BC is a highly complex multistage process that needs to be completed by cancer cells after they depart from the primary site to successfully spread and colonize secondary sites [99,100]. Since metastatic BC is the main cause of BC-related deaths, the metastatic cascade has been intensively studied to elucidate all the responsible mechanisms and factors and develop targeted drugs to inhibit various stages of the process. The epithelial-to-mesenchymal transition (EMT) is an essential process in BC metastasis as by undergoing this transition, epithelial cancer cells gain invasiveness and migratory potential, characteristics that promote intravasation and improve the cell survival potential in circulation [101,102,103]. Following EMT, CTCs gain mesenchymal attributes and, as a result, the expression of mesenchymal proteins (N-cadherin, fibronectin, and vimentin) is upregulated. In contrast, the expression of specific epithelial molecules such as EpCam, E-cadherin, claudins, and cytokeratins is downregulated as CTCs lose their epithelial phenotype [104,105]. However, not all the CTCs that detach from a tumor and are shed into circulation possess the ability to populate distant tissues and organs and generate secondary metastases, but the shift from an epithelial to a mesenchymal phenotype improves tumor cells’ stability in circulation. Moreover, CTCs detach and travel in circulation as individual cells or grouped in clusters, features that increase their metastatic potential and stability in the bloodstream [106]. The clusters can be either formed exclusively by tumor cells (homoclusters) or after interaction with other blood-circulating cells (heteroclusters) [107,108]. However, not all the CTCs that detach from a tumor possess the ability to populate distant tissues and organs and generate secondary metastases because, in circulation, the CTCs’ fate is still challenged by both physical and biological threats. The vast majority of CTCs die due to anoikis, hemodynamic forces triggering apoptosis, or attack of the immune system [109], leading to a low yield of extravasated CTCs that will finally generate secondary metastases. Preclinical studies have revealed that less than 0.1% of CTCs remain viable in circulation and, finally, only 0.01% of these invade distant organs and generate metastasis [110]. Besides survival in circulation, for metastasis formation, CTCs are required to regain their epithelial phenotype by following the reverse EMT, the mesenchymal-to-epithelial transition (MET) [111].

As LB components, CTCs have been extensively studied as clinical biomarkers in BC, but their genetic and phenotypic heterogeneity, together with the low number of cells in blood samples, are challenges for accelerating the use of CTCs in BC management. Compared with blood cell numbers (5–9 × 10^9^/mL red blood cells, 5–10 × 10^6^/mL white blood cells, and 2.5–4 × 10^8^/mL platelets), CTCs are considered ultrarare events with a ratio of 1 CTC to 10^6^–10^7^ leukocytes in the peripheral blood of cancer patients [112,113]. The number of CTCs in blood samples is dependent on the cancer stage, with 70% of metastatic BC presenting 1CTC/7.5 mL of blood, numbers that highlight the need for sensitive detection methods for CTC isolation and analysis, especially for early BC [114]. As CTCs’ enumeration and molecular characterization (DNA, RNA, and protein levels) hold great promise in cancer management, numerous detection and analysis methods have been developed, but the majority have not fully validated their clinical utility. Moreover, if viable CTCs are retrieved following the isolation procedure, functional tests using cell cultures or CTC-derived xenografts can be performed [115]. To overcome CTCs’ extreme rarity, the techniques employed for CTC detection and analysis are preceded or include an enrichment strategy that is based either on tumor cells’ specific physical characteristics or their unique biological signature [116]. Based on the different physical properties of CTCs and blood cells, their size, density, deformability, and electrical properties have been explored for CTC capture [113].

Immunocapturing CTCs using magnetic immunoaffinity labeling is the basis of the majority of existing CTC isolation techniques. Considered a positive enrichment strategy, tumor cells are selected from blood samples with the help of CTC-specific antibody-coated beads and sequentially isolated by applying an external magnetic field [117,118]. For example, the only technology cleared by the Food and Drug Administration (FDA) for CTC detection and enumeration in cancer patients with metastatic breast, prostate, and colorectal cancer follows this enrichment strategy [119,120,121]. In the CellSearch platform, EpCam-positive cells (EpCam+) are magnetically captured through ferrofluidic nanoparticles functionalized with EpCam antigen. To validate the captured cells as CTCs, the captured cells are stained with cytokeratin monoclonal antibodies, CD45 monoclonal antibodies, and DAPI to highlight cell nuclei. Finally, only cells that are cytokeratin+/DAPI+/CD45- are validated as CTCs and added to the CTC count to determine the patient’s prognosis [122]. However, the clinical utility of the CellSearch platforms remains controversial, as the detection method is exclusively based on EpCam expression. Due to the EMT transition, CTCs can present low or no EpCam expression, and therefore these cells are not captured by EpCam-based methods [121]. Using a cancer-specific cocktail of antibodies for CTC capture or a negative enrichment strategy to remove blood cell components can address these limitations and capture all subpopulations of CTCs [123,124,125]. Despite these observations, EpCam still represents the backbone of CTC capturing and detection methods, especially for cancers that are associated with a strong expression of EpCam, including BC. For BC, specific molecular markers such as HER2 and ER have been successfully employed for CTC detection, even in patients with early BC [126,127,128,129].

Depending on the disease stage and detection technology, CTCs are routinely detected in 10–80% of BC patients [130], with a significantly higher rate of detection in metastatic BC cases. Therefore, CTCs showed great potential as biomarkers for prognosis, monitoring therapeutic response, and recurrence risk stratification. For diagnosis and early detection of BC cases, CTCs are still being explored since the limited sensitivity of the available CTC detection methods hinders their effective use. For example, Karimi et al. screened the capacity of Magnetic Activated Cell Sorting (MACS) to identify CTCs in the blood of treatment-naive BC patients in various stages using EpCam and a panel of cytokeratins (CK) for detection (CK7, CK8, CK18, and CK19) [131]. Their study revealed the limitations of CTCs for BC detection as CTCs were not detected in blood samples of stage I BC patients but showed the utility of using CTCs as a prognostic and therapy modulation tool. In contrast, promising results were presented by CTC capturing and enumeration using a nanostructured titanium oxide-coated slide, as CTCs were identified in early-BC stages [132].

The identification of CTC detection and enumeration as a powerful tool for BC patients’ prognosis is sustained by numerous studies. CTC enumeration serves as an independent prognostic marker in BC, with a higher CTC count being associated with adverse clinical outcomes, including disease recurrence and metastasis development [133]. Numerous studies have demonstrated that the presence of CTCs in the peripheral blood of BC patients is correlated with poor OS and reduced disease-free survival rates [133,134,135]. Moreover, the dynamic changes in CTCs may represent a surrogate prognosis biomarker in cancer progression, and their enumeration during the course of treatment also provides valuable insights into the treatment response and emergence of resistant clones [136]. When monitoring the therapy response, a decrease or clearance in CTC count is associated with a good therapeutic response, while an increase in CTC count is an alarming signal that the therapeutic regimen is not effective [137,138].

### 4.2. Circulating Tumor Nucleic Acids (ctNAs)

ctNAs represent only a small fraction of the total circulating cell-free nucleic acids detected in circulation and comprise different subtypes of molecules, with circulating tumor DNA (ctDNA), and circulating tumor microRNAs (miRNAs) being the most frequently explored as LB analytes. Despite the shift in LB research from CTCs towards ctNAs, these two classes of biomarkers are distinct and provide different information and often cannot be simultaneously detected in patients’ blood samples. For example, ctDNA was present in blood samples from patients without detectable CTCs [148].

The presence of cell-free DNA (cfDNA) in circulation is not reported only in cancer patients. From the total cfDNA released from both tumor and healthy cells in circulation by apoptosis, necrosis, or active section, only a small fraction of degraded fragments of DNA are identified as ctDNA (<0.1–<5% of the total cfDNA) [149]. The extent of ctDNA fragmentation is influenced by the release mechanism, with necrosis releasing longer fragments compared to those released by apoptosis [150,151]. Moreover, the concentration of ctDNA in the blood is dependent on cancer type and stage, but in advanced cancer cases, the cfDNA concentration is significantly increased compared with healthy controls, whereas for early-stage and non-metastatic patients, ctDNA represents between 0.1 and 1% of the total cfDNA [152]. Based on its relatively short life in circulation (15 min–2 h), ctDNA is also considered a cancer dynamic biomarker, therefore being employed for real-time tumor monitoring [153].

ctDNA research holds great promise in unraveling the tumors’ genetic makeup by allowing the identification of different genomic alterations by sensitive molecular assays using PCR-based or NGS-based techniques. ctDNA is characterized by genetic and epigenetic alterations such as point mutations, copy number variations (CNVs), chromosomal rearrangements, or specific methylation patterns [154]. PCR-based methods such as digital droplet PCR (ddPCR), BEAMing (beads, emulsion, and amplification technology), or ARMSs (amplification refractory mutation systems) showed appropriate sensitivity in ctDNA detection in BC ranging from 0.1% to 0.001% [155,156,157,158,159,160] and are definitively less costly than NGS assays. The main disadvantage of PCR-based methods is that targeted genetic or epigenetic alterations must be known beforehand. Therefore, PCR-based assays are limited to either BC hotspot mutations (PIK3CA, TP53, PTEN, AKT1, etc.) or require a preliminary tumor analysis to reveal predetermined mutations [161]. In addition, PCR-based methods such as methylation-specific PCR (MS-PCR) could reveal methylation patterns in ctDNA including the methylation of promoters and enhancers, processes that play a critical role in the regulation of gene expression and are implicated in BC development and progression [162]. NGS technology addresses these limitations and captures the BC tumor molecular landscape with superior sensitivity [163,164], either by a targeted approach to simultaneously investigate a relevant panel of genes in BC or whole-genome sequencing that can help to identify novel molecular matched therapies.

The opportunity to perform repetitive blood sampling at specific time points during the course of BC development allowed the exploration of ctDNA as a biomarker for diagnosis, prognosis, therapy modulation, and recurrence risk prediction, but it remains to be discussed whether this analyte can be used for early BC cases, especially due to the low concentrations found in the bloodstream. For patients with mBC, ctDNA is detectable in the majority of the cases [165]. For these patients, the European Society of Medical Oncology (ESMO) recently recommended the routine use of ctDNA assays in clinical practice, citing ample evidence that these tests are effective for tumor genotyping to guide molecularly targeted therapies in patients with metastatic cancer [166].

Despite the low concentration of ctDNA in blood samples of early BC, promising results were obtained by screening this circulating biomarker for early diagnosis. Rodriguez et al. used Safe-Seq to detect mutations in patients with unusual mammography findings before they underwent tissue biopsy and revealed that ctDNA could be employed for early BC diagnosis, finding four mutations (three in TP53 and one in PIK3CA) only in plasma samples, showing that liquid biopsies could capture the tumor heterogeneity [167]. Moreover, ctDNA was successfully employed for monitoring MRD as ctDNA detection after surgery and could predict relapse months before clinical signs appeared. The presence of ctDNA after surgery was associated with a significantly higher risk of relapse, making it a powerful circulating marker for patient risk stratification [168]. In another study [169], ctDNA proved to be useful for identifying BC patients at high risk of recurrence, with plasma ctDNA being identified prior to clinical or radiological signs in 16 out of 18 patients who eventually relapsed, yielding a sensitivity of 89%. None of the 31 patients who did not relapse tested positive for ctDNA at any time across 156 plasma samples, indicating a specificity of 100%. Among the two relapsed patients who were not detected by ctDNA analysis, one experienced only a local recurrence, and the other had a bone recurrence and had completed chemotherapy merely 13 days before the blood sample was taken.

On the other hand, circulating miRNAs are short non-coding RNA molecules (20–25 nucleotides) that play crucial roles in post-transcriptional gene regulation by targeting messenger RNA (mRNA) for degradation or translational repression [170,171,172,173]. Following transcription in the nucleus, miRNAs are transported to the cytoplasm, where they undergo maturation into mature miRNAs. miRNAs were identified in all body fluids, either in free form or encapsulated within extracellular vesicles that protect them. miRNA deregulation plays a crucial role in the pathogenesis of BC, as altered patterns of miRNAs are associated with tumor cell proliferation, invasion, migration, and chemoresistance [174,175]. Moreover, variations in the miRNA levels are indicative of various conditions of the body, including BC. Based on their role in cancer, miRNAs can exert either oncogenic or tumor-suppressive effects by regulating the expression of the target gene, meaning it is categorized as oncomiRs or tumor-suppressor miRNAs [174,175]. For example, miR-21 is usually overexpressed in cancer patients with BC, with high levels of miR-21 associated with a poor prognosis, advanced clinical stage, lymph node metastasis, and lower OS rates [176]. Similarly, the expression of miR-155 is upregulated in BC cases, associated with aggressive tumor phenotypes, advanced stages of disease, and a poor prognosis [177]. Khalighfard and collaborators [178] proposed a panel of oncomiRs (miR-21, miR-155, and miR-10b) and tumor-suppressor miRs (Let-7a) to monitor non-metastatic BC patients after operation, chemotherapy, and radiotherapy. Their results revealed an initial significant increase in miR-21, miR-155, and miR-10b in BC patients’ plasma samples as compared with healthy individuals, with matching expression found in tissue samples. Moreover, comparing the levels of circulating miRs after operation, chemotherapy, and radiotherapy revealed a reduction in oncomiR expression and an increase in tumor-suppressor miRs, highlighting the potential of these circulating biomarkers for the real-time monitoring of BC patients. In the search for other markers to enable a better prognosis of BC patients and risk stratification, miR-200c and miR-34a have also been explored as their reduced levels predict poor patient survival, making them appealing as novel therapeutic approaches [179]. Moreover, miRNAs are useful in predicting response to therapy, with miR-221 being used as an indicator of BC resistance to endocrine therapy as increased levels have been associated with BC patients’ resistance to tamoxifen and fulvestrant therapy [180], while miR-155 and miR-31 are linked to chemoresistance [181,182].

### 4.3. Tumor-Educated Platelets (TEPs)

Blood platelets, small anucleated cells derived from megakaryocytes cytoplasm, typically survive in circulation for 8–11 days [183,184]. While primarily recognized for their critical role in regulating homeostasis and thrombosis, recent research has revealed their secondary function of facilitating tumor progression and metastasis by their cross-talk with tumor cells and their involvement in the angiogenic process in the metastatic niche [185,186]. Moreover, platelets have been observed to ingest cellular RNA, either during circulation or following interaction with other cell types, including tumor cells [187,188]. The TEPs arise from the direct or indirect interaction between tumor cells and platelets, triggering alterations in the RNA and protein cargo of the platelets. For example, cancer RNA biomarkers such as KRAS, PCA3, PIK3CA mutants, EGFRvIII, FOLH1, KLK2, KLK3, EML4-ALK, and NYP have been reported to be sequestered by platelets that actively uptake tumor-derived material [189].

TEPs have been explored as liquid biomarkers for multiple purposes such as BC diagnosis, prognosis, prediction, and cancer surveillance. Independent of the downstream application, one key aspect of using TEPs in LB is the protocol used for platelet isolation from blood samples due to their fragility and the risk of activating them during sample processing protocols. At the moment, the most commonly employed method for platelet separation is based on low-speed centrifugation of the anticoagulated blood samples, and it has been proven to be an effective method to recover a high-purity fraction of platelets, with minimal activation observed [190,191]. For example, Best et al. [190] studied 283 platelets samples including 39 BC-derived samples and revealed the utility of TEP-based liquid biopsies to guide clinical diagnosis and therapy selection. Their study revealed that TEPs provide a useful RNA biosource, as healthy individuals can be discriminated from cancer patients with 96% accuracy using their mRNA expression profiles. Moreover, the location of the primary tumor can be determined with high accuracy (71%), while TEP mRNA profiles can be useful to highlight HER2-positive, PIK3CA, and triple-negative BC tumors. While their promising results suggested that TEPs can be employed as a companion diagnosis tool, Liefaard et al. pointed out the need to further improve the TEP protocols for their prospective use for diagnosis purposes, showing that in a single-center, independent, blinded study, the gene expression of TEPs was heavily influenced by the sample’s hospital of origin and other factors [192].

## 5. Conclusions and Perspectives

In summary, the advancements in molecular profiling have significantly enhanced the understanding and management of BC. Traditional diagnostic methods, while foundational, have been complemented by innovative techniques such as NGS and liquid biopsy. These methods offer comprehensive insights into the genetic and molecular landscape of BC, enabling more precise and individualized treatment approaches.

The integration of molecular profiling technologies, including circulating tumor cells (CTCs), circulating tumor DNA (ctDNA), and tumor-educated platelets (TEPs), has improved the accuracy of BC diagnosis and prognosis. These biomarkers provide valuable real-time information about tumor dynamics and treatment responses, together with a better prognosis and disease recurrence identification. The identification of specific molecular subtypes through advanced profiling allows for the development of tailored therapies, improving treatment efficacy and minimizing adverse effects, thereby leading to better patient outcomes. Additionally, liquid biopsy techniques, especially the analysis of ctDNA and CTCs, offer a non-invasive method to monitor disease progression and therapeutic responses continuously. This real-time monitoring capability is crucial for the early detection of relapse and adjustment of treatment plans. Despite the promising advancements, there are challenges to be addressed, including the need for standardization in biomarker detection methods, the high costs associated with advanced technologies, and the variability in the sensitivity and specificity of different assays.

Looking forward, further research is essential to validate and refine the use of molecular profiling technologies in clinical practice. Studies focusing on the longitudinal analysis of ctDNA and CTCs could provide deeper insights into tumor evolution and resistance mechanisms. Emerging technologies such as single-cell sequencing and spatial transcriptomics hold great potential for uncovering new biomarkers and therapeutic targets. To maximize the clinical benefits, there is a need to develop cost-effective and accessible molecular profiling methods. Efforts should be directed toward simplifying the technology and making it available in low-resource settings. The successful implementation of these advanced techniques requires collaboration among oncologists, pathologists, bioinformaticians, and other healthcare professionals. This interdisciplinary approach will ensure that molecular profiling is effectively integrated into routine clinical practice.

In conclusion, molecular profiling represents a paradigm shift in the management of breast cancer. By enabling more precise diagnostics, personalized therapies, and real-time monitoring, these advancements promise to significantly improve patient outcomes. However, addressing the existing challenges through continued research and technological innovation is crucial for realizing the full potential of molecular profiling in clinical oncology.

## Figures and Tables

**Table 1:** List of targeted therapy drugs approved for BC therapy, providing the generic drug name and brand name.

Therapeutic Setup: HER-2 Positive BC		
Type	Monoclonal antibodies	Agents: Trastuzumab (Herceptin), Pertuzumab (Perjeta), Ado-trastuzumab emtansine (T-DM1, Kadcyla), Pertuzumab, trastuzumab, Trastuzumab deruxtecan (Enhertu) and hyaluronidase-zzxf (Phesgo)
	Tyrosine kinase inhibitors	Agents: Neratinib (Nerlynx), Lapatinib (Tykerb), Tucatinib (Tukysa)
	Antibody-Drug Conjugates	Agent: Margetuximab-cmkb (Margenza)
Therapeutic setup: Hormone receptor-positive BC		
Type	Selective Estrogen Receptor Modulators (SERMs)	Agents: Tamoxifen citrate (Soltamox), Toremifene (Fareston)
	Selective Estrogen Receptor Degraders (SERDs)	Agents: Fulvestrant (Faslodex) Elacestrant dihydrochloride (Orserdu)
	Aromatase Inhibitors	Agents: Anastrozole (Arimidex), Letrozole (Femara), Exemestane (Aromasin)
	CDK4/6 Inhibitors	Agents: Palbociclib (Ibrance), Ribociclib (Kisqali), Abemaciclib (Verzenio)
Therapeutic setup: Triple-Negative Breast Cancer (TNBC)		
Type	Immune Checkpoint Inhibitors	Agents: Atezolizumab (Tecentriq), Pembrolizumab (Keytruda)
	Antibody-Drug Conjugates	Agent: Sacituzumab govitecan (Trodelvy)
Other indications:		
	PARP Inhibitors (for BRCA-Mutated Breast Cancer)	Agents: Olaparib (Lynparza), Talazoparib (Talzenna)
	PI3K Inhibitors	Agent: Alpelisib (Piqray)
	mTOR Inhibitors	Agent: Everolimus (Afinitor)
	AKT inhibitors	Agent: Capivasertib (Truqap)
	Antibody-Drug Conjugates	Agent: Trastuzumab deruxtecan (Enhertu)
